# Indication-wide drug pricing: Insights from the pharma market

**DOI:** 10.1186/s40545-022-00451-x

**Published:** 2022-08-29

**Authors:** Florian Siegmeier, Melanie Büssgen

**Affiliations:** Hamburg Center for Health Economics, University of Hamburg, Hamburg, Germany

**Keywords:** Pharmaceutical pricing, Pharma market, Reference pricing, Pharmaceuticals, Health policy, Price regulation, Market access, Pricing

## Abstract

**Background:**

Pharmaceutical spending has been increasing rapidly for years and is higher than ever before. To control the rising costs, countries are implementing regulatory frameworks such as (internal) reference pricing, price cuts or generics substitution. Internal reference pricing establishes a reference price within a country which serves as the maximum level of reimbursement for a group of pharmaceuticals. Price setting in the German market is especially relevant for many European countries, which use Germany as a reference country for their own price setting.

**Methods:**

We evaluate pharmaceutical price dynamics for not reference priced pharmaceuticals (NRPs) as well as for reference priced pharmaceuticals (RPs) in Germany—referring to the internal reference price system. 64,862 medication packs have been extracted from the German pharmaceutical pricing register Lauer-Taxe. For each pack, we extracted detailed data on the company, manufacturer rebates, pharmacy retail prices, reference prices, co-payments, import quotas, and discount agreements. We then investigated price setting and dynamics of NRPs vs. RPs for all 14 indication areas by ATC code level 1.

**Results:**

The average manufacturer price per pack was 604.84€ for NRPs and 112.11€ for RPs. Similar differences were found for the wholesale price and the pharmacy retail price. The reference price was—as expected—0.00€ for NRPs, and 154.40€ for RPs. NRP packs were imported in 42.38%, while RP packs were imported only in 24.62%. Highest average pharmacy retail prices could be found in the therapeutic areas ‘antineoplastic and immunomodulating agents’ (1711.47€), ‘systemic hormonal preparations’ (1331.95€), and ‘blood and blood forming organs’ (1260.58€). We detected high fluctuations in pharmacy retail prices per indication, as well as for reference prices per indication. The indications with the highest number of reference price regulated medical packs are ‘cardiovascular system’, ‘musculo-skeletal system’, and ‘nervous system’. Highest co-payments were found in the indications ‘antineoplastic and immunomodulating agents’, ‘blood and blood forming organs’, and ‘antiinfectives for systemic use’.

**Conclusion:**

Price setting and price dynamics vary substantially between NRP and RP medication packs. Further, we saw major differences across all indication areas as well as when comparing medication packs launched by top 20 pharma companies vs. the rest.

**Supplementary Information:**

The online version contains supplementary material available at 10.1186/s40545-022-00451-x.

## Background

Health care expenditure has been increasing rapidly for years and is higher than ever before [1]. Over the last two decades health care spending around the world rose from 8.7% of GDP in 2000 to 9.9% of GDP in 2018 [2]. Especially new pharmaceuticals are costs drivers in health care systems, reaching a new peak of $1.4 trillion worldwide [3]. To control the rising pharmaceutical costs, countries are implementing regulatory frameworks such as reference pricing, public tendering, price cuts and discounts, cost-sharing models, prescription guidelines for physicians, margin regulation or generics substitution [4]. In Germany, the pharmaceutical market restructuring act (AMNOG) was introduced in 2011, ensuring pharmaceutical innovation and economically efficient prices. The regulation is a two-stage approach in which price negotiations refer to evidence-based medical benefit assessments from prior clinical trials [5]. Drugs with a proven additional benefit could negotiate a higher price than the price of the current standard of care. In principle, it was found that the greater the proven additional benefit, the higher the negotiated price for the new authorised drug [6]. Drugs without additional clinical benefit will be sorted into a reference price group.

Germany was the first country to implement internal reference pricing (IRP) in 1989 to protect the insured community from excessive drug prices [7]. The fixed price of a medicinal product is the maximum amount that the statutory health insurances (SHI) pay for this pharmaceutical. If its sales price is higher than the fixed price, patients usually either pay the difference to the fixed price themselves or receive another—therapeutically equivalent—drug without additional payment [8]. The German Pharmaceutical Market Restructuring Act (AMNOG) confirms the important price control function of the reference price system, but also creates a regulatory framework that regulates the benefit assessment of new medicines more clearly than before and introduces a new SHI reimbursement amount for innovative medicines [7]. New patent-protected pharmaceuticals that represent a therapeutic improvement, e.g. due to fewer side effects, are excluded from the fixed-priced system [9].

Reference price systems are used in a number of European countries leading to price reductions of all pharmaceuticals included, being larger for originators than generics, and fostering price competition within the market [10]. Even for innovative drugs, such as biologics and biosimilars, reference prices have a huge cost-containment potential as well as positive prescription effects [11]. Prior research has also found that the affordability of pharmaceuticals varies through indication areas [12–16]. Moye-Holz and Vogler investigated the price setting of cancer drugs in 16 European and Latin American countries and showed that high-income countries report lower PPP-adjusted prices for cancer medicines than middle-income countries and therefore were more affordable [17]. Morgan (2020) argues that many prices for pharmaceuticals, especially for rare diseases, exceed value for money thresholds and affordability in North America and are not justified by research and development costs at all [18].

Price setting in the German market is especially relevant for many European countries, which use Germany as reference country for their own price setting [19]. An impact analysis of cross-reference pricing in the former EU-15 countries showed that for a price reduction of 1.00€, EU-15 reimbursement prices dropped in a range of 0.15€ (Austria) to 0.36€ (Italy) [20].

However, indication-wide price setting and price dynamics of not reference priced pharmaceuticals as well as reference priced pharmaceuticals have been an under-researched area so far. With this article, we provide an extensive overview of pharmaceutical pricing across all 14 indication areas by ATC code level 1. Our analysis encompasses an evaluation of pharmacy retail prices, reference prices, co-payments, import quotas as well as discount agreements. To the authors’ knowledge, this is the first study that provides a snapshot of pharmaceutical pricing and price dynamics across all 14 indication areas in the German pharma market.

## Methods

Medication packs have been extracted from the German pharmaceutical pricing register Lauer-Taxe in June 2021 [21]. For each medication pack, we extracted detailed data on (I) the company, (II) manufacturer rebate, (III) pharmacy retail price, (IV) Taxe purchasing price, (V) Taxe selling price, (VI) reference price, (VII) ATC code, (VIII) co-payment price, (IX) additional payment status [yes/no], (X) import status [yes/no], and (XI) discount agreement status [yes/no]. The (VI) reference price was defined as maximum amount paid by the statutory health insurances for a pharmaceutical in the reference price system.

Variables have been chosen as they are an effective tool for controlling prices and/or showing price fluctuations and dynamics in pharmaceutical markets [22–24]. As ‘price’ is not equal to ‘price’, we differentiated between ‘pharmacy retail price’, ‘Taxe purchasing price’, ‘Taxe selling price’, and ‘reference price’ to provide an overview of the different types of prices and also to investigate if differences between these types of prices could occur.

We restricted the sample to prescription pharmaceuticals. Further, we defined ‘indications’ as therapeutic areas and differentiated between all 14 therapeutic areas by ATC code level one for an indication-wide overview [25]: (1) alimentary tract and metabolism (META); (2) antiinfectives for systemic use (INFEC); (3) antineoplastic and immunomodulating agents (ONCIM); (4) antiparasitic products, insecticides and repellents (PARA); (5) Blood and blood forming organs (HAEM); (6) cardiovascular system (CARD); (7) dermatologicals (DERMA); (8) genito-urinary system and sex hormones (URO); (9) musculo-skeletal system (MUSCO); (10) nervous system (NEURO); (11) respiratory system (RESP); (12) sensory organs (SENS); (13) systemic hormonal preparations, excluding sex hormones and insulins (HORM), and (14) various indications (VAR). A list of the investigated indication areas can be found with single-digit letter abbreviation and indication abbreviation in the Additional files 1 and 3.

We further differentiated packs from the top 20 pharma companies (by revenue in 2020) [26, 27] vs. the remaining companies to see if differences in price setting occur. A list of the investigated top 20 pharma companies can be found in Additional file 2.

To investigate indication-wide price settings and price dynamics in Germany, we then calculated the mean of a medication pack’s reference price, the average pharmacy retail price, the pharmacy retail price for packs launched by top 20 pharma companies, the pharmacy retail price for packs launched by the remaining companies, the manufacturer rebate, and the amount of a co-payment, for each of the 14 indication areas. To detect differences between not reference priced (NRP) packs and reference priced (RP) packs, we run analyses twice—for NRP and RP packs. We also calculated a deviation factor between the NRP packs and RP packs to see differences more clearly. Furthermore, we calculated a deviation factor for reference price regulated pharmaceuticals which indicates the deviation of pharmacy retail prices (PRP) to the reference price (RP).

Additionally, we calculated the mean percentage of packs that (a) are reference price regulated; (b) have a discount agreement; (c) have no co-payment; (d) are below 30% of the reference price level (which should make them free from co-payment), and (e) are imported packs, for all 14 indication areas. Again, to detect differences between not reference priced (NRP) packs and reference priced (RP) packs, we run analyses twice—for NRP and RP packs.

We further generated graphs showing the average pharmacy retail price and the average reference price per indication, as well as the co-payment for NRP packs vs. RP packs.

All analyses were performed using STATA SE 16.

## Results

Our final sample included 64,862 medication packs, 27,618 of which were not reference price regulated, and 37,244 of which were reference price regulated.

Not reference price regulated packs (NRP) had an average manufacturer rebate of 42.80€, while reference price regulated packs (RP) had an average manufacturer rebate of only 0.56€. The average manufacturer price was 604.84€ for NRPs and 112.11€ for RPs. Similar differences were found for the wholesale price (609.09€ for NRPs vs. 115.63€ for RPs) and the pharmacy retail price (747.97€ for NRPs vs. 151.90€ for RPs). The reference price was -as expected- 0.00€ for NRPs, and 154.40€ for RPs. The average co-payment was 7.79€ for NRPs, while it was 5.23€ for RPs.

NRP packs had in 20.08% a discount agreement, while 37.63% of the RP packs had a discount agreement. In 6.24% NRP packs were exempt from co-payment, while 10.98% of RP packs were exempt from co-payment. 8.62% of NRP packs were launched by top 20 pharma companies, while it was only 4.69% for RP packs. NRP packs were imported in 42.38%, while RP packs were imported only in 24.62%. Table 1 reports the descriptive characteristics of the data set.

**Table 1:** Sample descriptives

Variable	Not reference price regulated (NRP) packages			Reference price regulated (RP) packages		
	N	Mean [€]	Mean [%]	N	Mean [€]	Mean [%]
Company	665			269		
Medication packs	27,618			37,244		
Molecule	2,051			635		
ATC level 1—indications	14			14		
Manufacturer rebate		42.80			0.56	
Manufacturer price		604.84			112.11	
Wholesale price		609.09			115.63	
Pharmacy retail price		747.97			151.90	
Reference price		0.00			154.40	
Co-payment		7.79			5.23	
Discount agreement			20.08			37.63
No co-payment			6.24			10.98
Top 20 pharma companies			8.62			4.69
Import			42.38			24.62

**Fig. 1:**
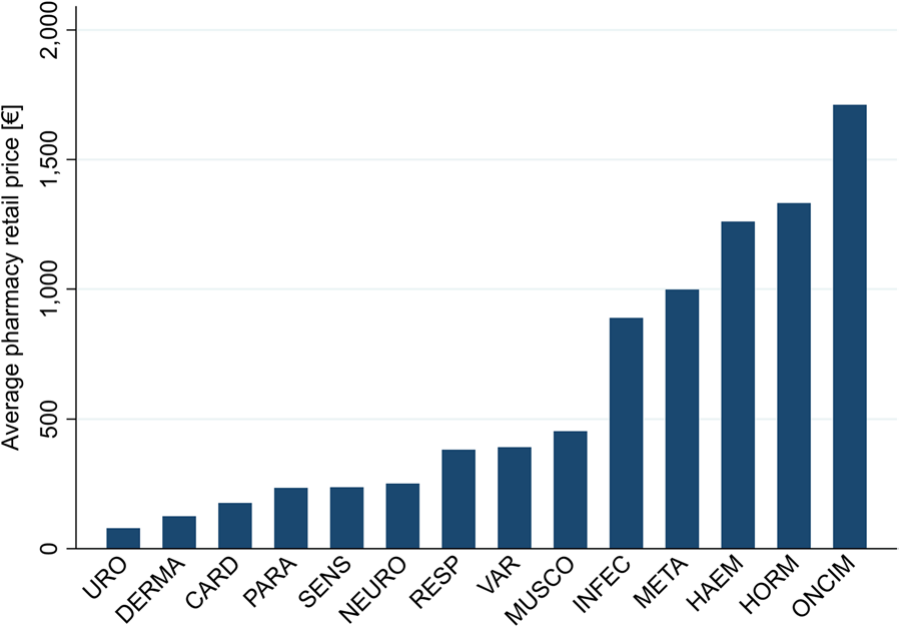
Average pharmacy retail price per indication, for not reference priced packs

Highest average pharmacy retail prices for NRPs could be found in the indication areas antineoplastic and immunomodulating agents (1711.47€), systemic hormonal preparations (1331.95€), blood and blood forming organs (1260.58€), alimentary tract and metabolism (998.05€), as well as antiinfectives for systemic use (888.76€). All other indication areas had average pharmacy retail prices of below 500.00€ (Fig. 1).

**Fig. 2:**
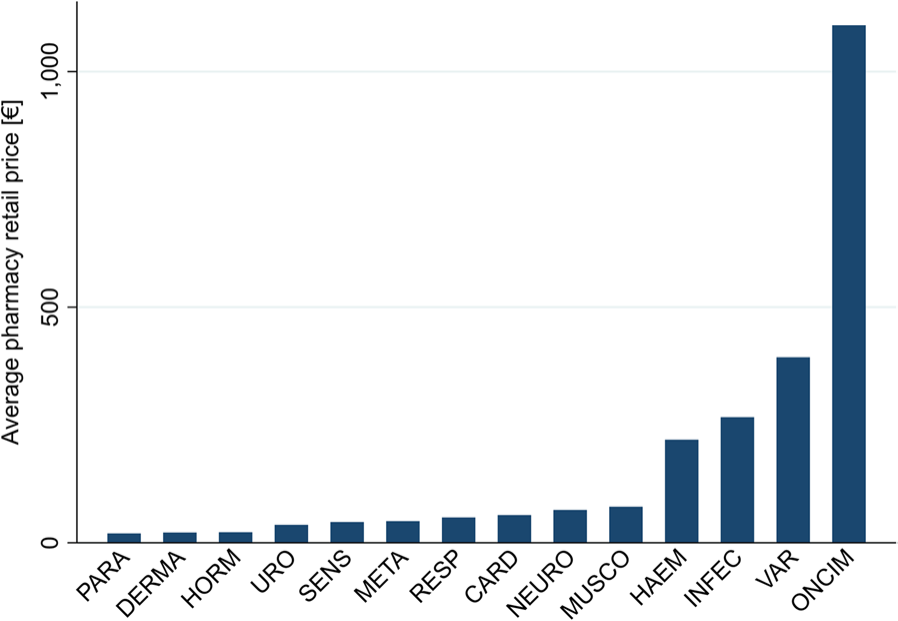
Average reference price per indication, for reference priced packs

For reference priced packs, we found that antineoplastic and immunomodulating agents still had the highest average pharmacy retail price (1098.39€). Substances that could not be assigned to any of the indication areas and were listed as "various" had the second highest average pharmacy retail price (393.47€). While antiinfectives for system use (266.48€) and blood and blood forming organs (218.57€) had average pharmacy retail prices of around 250€ per pack, all other investigated indication areas were below 100.00€ (Fig. 2).

**Fig. 3:**
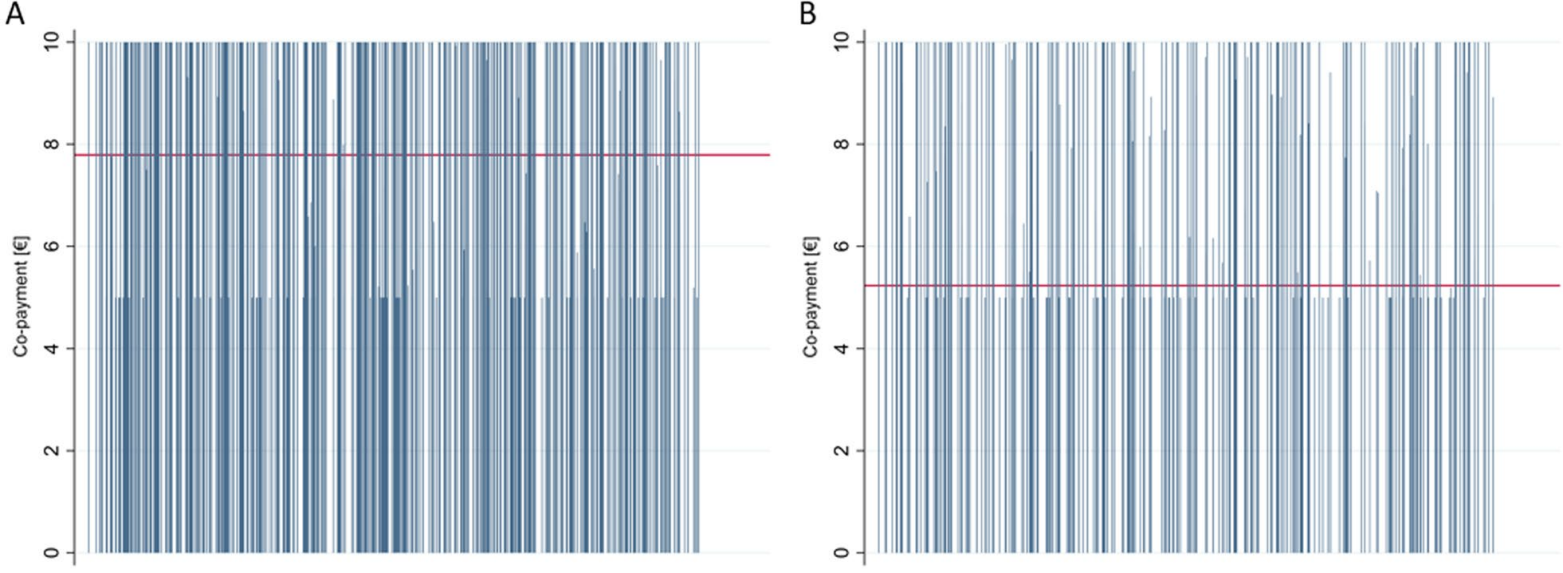
Average co-payment for NRPs (**A**) vs. for RPs (**B**)

Comparing average co-payments, we found that not reference priced packs had higher co-payments (7.79€) than reference priced packs (5.23€). Also, reference priced packs were more often exempt from co-payment (10.98%) than not reference priced packs (6.24%). From the graphs, however, it is noticeable that the 5.00€ co-payment has the densest graphical distribution (for both NRPs and RPs) (Fig. 3A and B).

We detected high fluctuations in pharmacy retail prices per indication (e.g. 79.16€ for URO vs. 1711.47€ for ONCIM), as well as for reference prices per indication (e.g. 20.16€ for PARA vs. 1098.20€ for ONCIM). Pharmacy retail prices of NRP packs were—as expected—significantly higher than pharmacy retail prices of RP packs (on average 604.84€ (NRPs) vs. 112.11€ (RPs)). Comparing NRP packs launches by top 20 pharma companies vs. the remaining companies, we found that while in most indication areas the packs from top 20 pharma companies were higher priced (e.g. INFEC: 1182.30€ (top 20 pharma companies) vs. 846.58€ (remaining companies), ONCIM: 2208.60€ (top 20 pharma companies) vs. 1645.24€ (remaining companies)), for some indications pharmacy retail prices were about the same (PARA: 246.24€ (top 20 pharma companies) vs. 233.29€ (remaining companies), URO: 75.22€ (top 20 pharma companies) vs. 79.25€ (remaining companies), SENS: 250.55€ (top 20 pharma companies) vs. 236.29€ (remaining companies)), and sometimes even lower (META: 592.76€ (top 20 pharma companies) vs. 1067.88€ (remaining companies), RESP: 238.22€ (top 20 pharma companies) vs. 406.45€ (remaining companies)). Comparing RP packs, we saw that pharmacy retail prices for the top 20 pharma companies are, in general higher, than packs from remaining companies, except for the indications ONCIM (910.66€ (top 20 pharma companies) vs. 1110.54€ (remaining companies)), MUSCO (62.21€ (top 20 pharma companies) vs. 76.93€ (remaining companies)), HORM (20.21€ (top 20 pharma companies) vs. 22.46€ (remaining companies)), and VAR (158.89€ (top 20 pharma companies) vs. 410.63€ (remaining companies)). We found very high deviation factors NRP–RP in the indication areas META (21.75x), PARA (11.92x), and HORM (60.05x), implying that for these indications the price differences between not reference priced packs and reference priced packs are the biggest. Looking now at the other investigated deviation factor PRP–RP, we saw that it revolves mostly between 1.0–1.5. If the pharmacy retail price is higher than the reference price for RP packs, it implies that the patient has to pay the remaining costs out-of-pocket. Packs with the highest manufacturer rebate were ONCIM (87.39€), HAEM (93.38€), and HORM (83.17€). Highest co-payment costs—and with that highest pack prices—were found in the indications INFEC (8.89€), ONCIM (9.79€), and HAEM (8.99€); see Table 2.

**Table 2:** Indication-wide average prices for not reference priced packs vs. reference priced packs

	META				INFEC				ONCIM				PARA				HAEM				CARD				DERMA			
	NRP		RP		NRP		RP		NRP		RP		NRP		RP		NRP		RP		NRP		RP		NRP		RP	
	€	dev. Factor NRP–RP	€	dev Factor PRP–RP	€	dev. Factor NRP–RP	€	dev Factor PRP–RP	€	dev. Factor NRP–RP	€	dev Factor PRP–RP	€	dev. Factor NRP–RP	€	dev Factor PRP–RP	€	dev. Factor NRP–RP	€	dev Factor PRP–RP	€	dev. Factor NRP–RP	€	dev Factor PRP–RP	€	dev. Factor NRP–RP	€	dev Factor PRP–RP
Reference price (RP)	–		45.09		–		254.55		–		1098.20		–		20.16		–		243.33		-		61.83		-		21.88	
Pharmacy retail price	998.05	21.75	45.88	1.018	888.76	3.34	266.49	1.047	1711.47	1.56	1098.39	1.000	234.39	11.92	19.66	0.975	1260.58	5.77	218.57	0.898	176.15	3.00	58.69	0.949	125.26	5.81	21.56	0.985
Pharmacy retail price, top 20 Pharma companies	592.76	10.45	56.71	1.258	1182.30	2.27	520.61	2.045	2208.60	2.43	910.66	0.829	246.24	9.61	25.63	1.271	3851.76	9.91	388.51	1.597	810.07	6.59	122.89	1.988	937.80	38.72	24.22	1.107
Pharmacy retail price, remaining companies	1067.88	23.57	45.30	1.005	846.58	3.31	255.69	1.004	1645.24	1.48	1110.54	1.011	233.29	12.27	19.01	0.943	855.65	4.10	208.49	0.857	118.52	2.10	56.36	0.912	102.78	4.81	21.39	0.978
Manufacturer rebate	50.80	175.17	0.29	–	44.43	17.99	2.47	–	87.39	46.48	1.88	–	14.66	97.73	0.15	–	93.38	274.65	0.34	-	6.27	16.95	0.37	-	6.28	29.90	0.21	-
Co-payment	7.76	1.45	5.34	–	8.89	1.46	6.08	–	9.79	1.12	8.75	–	7.34	1.57	4.67	–	8.99	1.42	6.34	-	7.71	1.64	4.71	-	6.36	1.36	4.68	-

	URO				MUSCO				NEURO				RESP				SENS				HORM				VAR			
	NRP		RP		NRP		RP		NRP		RP		NRP		RP		NRP		RP		NRP		RP		NRP		RP	
	€	dev. Factor NRP–RP	€	dev Factor PRP–RP	€	dev. Factor NRP–RP	€	dev Factor PRP–RP	€	dev. Factor NRP–RP	€	dev Factor PRP–RP	€	dev. Factor NRP–RP	€	dev Factor PRP–RP	€	dev. Factor NRP–RP		dev Factor PRP–RP	€	dev. Factor NRP–RP	€	dev Factor PRP–RP	€	dev. Factor NRP–RP	€	dev Factor PRP–RP
Reference price (RP)	–		38.71		–		80.02		–		72.16		–		58.29		–		46.39		–		22.61		–		421.19	
Pharmacy retail price	79.16	2.08	38.05	0.983	453.28	5.94	76.37	0.954	251.31	3.61	69.56	0.964	381.76	7.11	53.68	0.921	236.92	5.40	43.87	0.946	1331.95	60.05	22.18	0.981	391.50	0.99	393.47	0.934
Pharmacy retail price, top 20 Pharma companies	75.22	1.40	53.60	1.385	4663.41	74.96	62.21	0.777	329.84	3.76	87.71	1.215	238.22	4.14	57.55	0.987	250.55	5.18	48.33	1.042	1565.91	77.48	20.21	0.894	987.16	6.21	158.89	0.377
Pharmacy retail price, remaining companies	79.25	2.14	37.07	0.958	329.61	4.28	76.93	0.961	246.41	3.58	68.80	0.953	406.45	7.63	53.26	0.914	236.29	5.40	43.72	0.942	1308.50	58.26	22.46	0.993	373.54	0.91	410.63	0.975
Manufacturer rebate	5.15	24.52	0.21	–	25.52	55.48	0.46	–	12.47	43.00	0.29	–	16.70	66.80	0.25	–	11.95	51.96	0.23	–	83.17	519.81	0.16	–	50.30	13.00	3.87	–
Co-payment	6.40	1.48	4.31	–	8.37	1.50	5.59	–	8.02	1.40	5.73	–	7.74	1.38	5.61	–	6.25	1.11	5.63	–	8.81	1.74	5.07	–	8.18	0.92	8.87	–

NRP: not reference price regulated

RP: reference price regulated

Dev. Factor NRP–RP: deviation factor from not reference price regulated packs to reference price regulated packs

Dev. Factor PRP–RP: deviation factor from pharmacy retail prices to reference price

Looking now at how many packs are reference price regulated per indication, we found that the indications with the highest number of reference price regulated packs are CARD (84.11%), MUSCO (70.75%), and NEURO (74.60%). Rather low estimates were found for URO (29.89%) and VAR (3.98%). We found more discount agreements for RPs than for NRPs (e.g. META: 13.18% for NRPs vs. 30.76% for RPs, INFEC: 11.24% for NRPs vs. 42.34% for RPs, ONCIM: 28.59% for NRPs vs. 51.49% for RPs), except for HORM (42.19% for NRPs vs. 34.25% for RPs) and VAR (3.02% for NRPs vs. 0.00% for RPs). For NRPs, there was—as expected—no exemption from co-payment (for all indications: 0.00%), while estimates vary for RPs. While for some indications exemption from co-payment is rather high (INFEC: 14.32%, HAEM: 20.79%), for other indications it is rather low (ONCIM: 5.09%, SENS: 0.00%, VAR: 2.27%). Investigating how many packs are priced below the 30% RP level and should thus be exempt from co-payment, we found that for RP-regulated packs, the amount varies widely depending on the indication area (e.g. HAEM: 24.32% vs. HORM: 2.77%). For NRPs, estimates are—as expected—0.00% for all indications as those are not reference priced by definition. This implies that a higher percentage estimate for *below 30% RP level* than for *no co-payment*, would result in packs that are not exempt from co-payment now, but are generally eligible for exemption under the current German regulations. We also looked at import statistics and found that NRPs are usually more often imported than RPs (META: 44.84% for NRPs vs. 30.28% for RPs, INFEC: 50.40% for NRPs vs. 17.72% for RPs, HORM: 48.69% for NRPs vs. 4.28% for RPs), except for ONCIM (56.23% for NRPs vs. 62.86% for RPs), and HAEM (36.24% for NRPs vs. 71.47% for RPs). The indications with the highest import numbers are ONCIM (58.52%), HAEM (54.19%), and RESP (51.23%); see Table 3.

**Table 3:** Indication-wide price regulation details for not reference priced packs vs. reference priced packs

	META		INFEC		ONCIM		PARA		HAEM		CARD		DERMA	
	NRP	RP	NRP	RP	NRP	RP	NRP	RP	NRP	RP	NRP	RP	NRP	RP
	in %	in %	in %	in %	in %	in %	in %	in %	in %	in %	in %	in %	in %	in %
Reference price regulated	–	53.64*	–	42.28*	–	34.51*	–	36.52*	–	50.95*	–	84.11*	–	38.78*
Discount agreement	13.18	30.76	11.24	42.43	28.59	51.49	8.49	36.06	21.33	29.46	16.05	39.82	11.73	27.01
No co-payment	0.00	9.84	0.00	14.32	0.00	5.09	0.00	6.55	0.00	20.79	0.00	6.20	0.00	10.69
Below 30% RP level	0.00	8.54	0.00	18.66	0.00	8.66	0.00	8.19	0.00	24.32	0.00	15.81	0.00	11.07
Import	44.84	30.28	50.40	17.72	56.23	62.86	46.22	27.86	36.24	71.47	28.79	16.34	54.90	10.47

	URO		MUSCO		NEURO		RESP		SENS		HORM		VAR	
	NRP	RP	NRP	RP	NRP	RP	NRP	RP	NRP	RP	NRP	RP	NRP	RP
	in %	in %	in %	in %	in %	in %	in %	in %	in %	in %	in %	in %	in %	in %
Reference price regulated	–	29.89*	–	70.57*	–	74.60*	–	59.77*	–	53.80*	–	35.23*	–	3.98*
Discount agreement	20.45	37.35	26.17	37.29	24.97	37.62	14.86	30.01	11.09	28.11	42.19	34.25	3.02	0.00
No co-payment	0.00	7.96	0.00	6.20	0.00	10.69	0.00	7.96	0.00	0.00	0.00	1.76	8.16	2.27
Below 30% RP level	0.00	19.84	0.00	18.52	0.00	13.81	0.00	17.59	0.00	15.97	0.00	2.77	0.00	2.27
Import	32.70	20.45	39.57	21.62	39.01	16.02	63.25	43.15	44.36	46.08	48.69	4.28	10.19	0.00

NRP: not reference price regulated

RP: reference price regulated

^*^ referring to whole dataset

## Discussion

With this article, we provide an extensive overview of the German retail pharma market with its price setting and market dynamics. We conducted an analysis of all prescription pharmaceuticals listed in the German pharmaceutical price register in June 2021 to generate insights in pricing and cost-containment measures of pharmaceuticals across all ATC-1 indication areas. In general, pharmaceuticals not included in the German reference price system are higher priced, but fluctuations in pharmacy retail prices can be found across all indications.

The results indicate that reference pricing is a valid and useful tool for cost-containment in Germany. Comparing the ex-manufacturer price, wholesaler price and the pharmacy retail price, there is a difference of 80–81% between NRP and RP packs. However, the large price difference of approx. 80% between NRPs and RPs could be explained by the fact that RP packs are dominated by packs that are under generic competition and NRPs in fact having a higher additional benefit. Since 2011, new patent-protected medicines have had to go through the AMNOG procedure and are allowed to negotiate the reimbursement price with the SHIs if they can prove an additional benefit. It has already been found that drugs with a higher added benefit were also able to negotiate a higher price [28]. Nevertheless, reference pricing is a big cost-containment factor and saves the health insurance funds more than 8 billion € every year in Germany. Comparing this to rebate contracts (cost-containment of 5 billion € every year) and the AMNOG procedure (cost-containment of 3.6 billion € every year) reference pricing has the largest power to reduce drug prices [29, 30].

Additionally, in Germany there are RP packs in all indication areas. Admittedly, the proportion of RP packs on all packs in the various indication areas varies, but if “VAR” is excluded, the share of RP packs is at least 30% per the remaining indication areas. The highest proportion of RP packs can be found in the indication areas including common chronic diseases which are related to high long-term costs to the health care system (CARD 84%, MUSCO 71% and NEURO 75%) [31, 32].

Figure 2 points out the effect of reference prices across the various indication areas. In 10 of 14 indication areas, the average pharmacy retail reference price is below 100€. Furthermore, deviations from reference price were rather minor in both directions (factor 1.0 to 1.5 if retail price is above RP; 0.92 to 0.98 if retail price is below RP). Out-off-pocket payments are therefore rather low.

The highest retail prices can be found in indication areas with high costs of medical treatment [32]. Fluctuations in pharmacy retail prices can be explained by the German reimbursement mechanism. In the first-year post-launch, manufacturers are free to set the prices for newly launched pharmaceuticals. After the AMNOG assessment, reimbursement prices are set according to the added benefit and considering prices of comparable medicines [6]. Therefore, as a result, there are high price fluctuations across all indication areas.

NRP packs are—as predicted—significantly higher priced than pharmaceuticals in the reference price system. Comparing the deviations of the pharmacy retail price between NRP and RP packs it is not unexpected that NRP packs are more costly than RP packs, but not extensively. Only in the indication areas META (21.75x), PARA (11.92x), and HORM (60.05x) the deviation factor from reference price was significantly higher. Interestingly, pharmaceuticals of the top 20 pharma companies are higher priced than pharmaceuticals of the remaining sellers. This might be due to higher profit objectives and higher research and development costs as most of them are research-based pharma companies. Only in the minority of indication areas, products are priced about the same (e.g. PARA) or even lower (e.g. RESP).

In terms of affordability, our results show that, in accordance with Moye-Holz and Vogler 2021, antineoplastic and immunomodulating agents, which include oncologics, are the most expensive substances even if they are regulated through a reference price [17]. Affordability in general is a growing concern of health systems worldwide, as access to medicines creates social value but creates policy challenges due to the high costs involved as well [18]. The case of vaccines shows that the most widely used vaccines are the most expensive ones [33]. To ensure access to innovative and affordable pharmaceuticals, it is necessary to discuss new business models and to consider transparency measures as some pharmaceuticals are increasingly priced less affordable for payers [18, 33, 34].

Despite high average retail prices in the indication areas ONCIM (1711.47€), HORM (1331.95€), HAEM (1260.58€), META (998.05€) and INFEC (888.76€), medicines are generally affordable for every citizen in Germany, as in the majority of indication areas pharmaceuticals are priced on average below 500€. Furthermore, health insurance companies cover the expenses for all prescribed pharmaceuticals and having a health insurance is mandatory in Germany. Only a small co-payment between 5 and 10€ has to be payed to collect the prescribed medicine from the pharmacy. Since Germany is a reference country for other European countries, with different coverage schemes for pharmaceuticals, high prices for pharmaceuticals do have a direct impact, potentially making pharmaceuticals unaffordable in other countries [17, 19].

Interestingly, although pharmacy retail prices of a high number of RP packs (see Table 3) are below the potential exclusion margin of 30%, they are not exempt from co-payment by the GKV-SV even though they are eligible. The number of packs ≤ 30% varies between the indication areas, ranging between 2.27% (VAR) and 24.32% (HAEM), but only 11% of all RP packs are exempt from the co-payment. A reason might be, that the retail prices for RP packs are still not low enough to generate profitable savings for the GKV-SV. Therefore, an exemption from co-payment is not effective. Future research could investigate that in more detail.

Considering co-payments, our results show that the out-of-pocket payment for RP packs is distinctly lower than the co-payment for NRP packs. From the graphs A and B of Fig. 3, it is noticeable that the 5.00€ co-payment has the densest graphical distribution (for both NRP and RP packs), implying that medication packs usually cost between 50.00 and 100.00 € per pack.

Rebate contracts are more common for RP packs than for NRP packs, indicating that health insurances companies are eager to reduce pharmaceutical spending even if cost-containment measures are in place (i.e. reference price system). Discount agreements therefore play a major role in Germany’s reimbursement system. In case of parallel imports of pharmaceuticals, it is evident that the import rate is distinctly lower for RP packs than for NRP packs in all indication areas and overall (24.62% vs. 42.38%), indicating that the import of reference price medicines is less attractive and profitable due to lower pharmacy retail prices. However, it should be noted that RP packs are dominated by packs that are under generic competition. Thus, the fact that prices are lower for RP packs than for NRP packs and that rebate contracts are more common for RP packs than for NRP was to be expected. An explanation for the higher proportion of co-payment-free packs and the lower proportion of imported packs could be that rebate contracts are more common for RP packs.

However, there are also many studies that criticise reference pricing and consider it inappropriate for cost containment. E.g. it has already been found that while reference pricing produced substantial savings, the intensity of the impact differed between different pharmaceuticals. Thus, the additional cost savings from reference pricing after prior implemented generic substitution, are comparatively low [35]. Also, prior research found that—while the reference price decreases—affected firms increased their prices (particularly for off-patent branded products), which resulted in an increase in co-payment paid by patients. With high variation across treatments, such price effects resulted in a 17% decrease in branded medication consumption. Savings were then primarily obtained through increased patient co-payments. Furthermore, pharmaceutical companies' reactions to the reference price reduction were unexpected, implying underlying competitive dynamics that should be explored before policy changes [36].

### Limitations

Our analyses were subject to several limitations. First, our descriptive results are based on means which implies that potential outliers could have caused the mean to go up or down. However, double-checking this with the median, we see that the absolute values change, but the factors between RPs and NRPs remain the same.

Second, our measures could be biased by the number of packs per active substance and the large heterogeneity within one indication group (ATC-1 group). However, as the results across indications are in accordance with related literature, bias are expected to be rather small [37].

Third, our study focused only on German data. While we are able to examine all currently available medication packs on the German pharma market, we are not able to compare our results with price setting and dynamics in other countries. Thus, results are not generalisable to other countries.

Finally, as mentioned in the methods before, we report the current status of price levels and dynamics in Germany. As pricing and reimbursement schemes undergo regular small changes in Germany and the pharma market often follows trends, price trends and dynamics are likely going to change in the future depending on the prior factors.

## Conclusion

The findings of our study provide important evidence of the current price setting and price dynamics of not reference priced drugs as well as reference priced drugs in Germany, especially in terms of investigating different indications and companies. Prices vary significantly between not reference priced medication packs and reference priced medication packs. Further, we saw major differences across all indication areas as well as when comparing medication packs launched by top 20 pharma companies vs. the rest.

Highest average pharmacy retail prices could be found in the indication areas ONCIM, HORM, and HAEM. We detected high fluctuations in pharmacy retail prices per indication, as well as for reference prices per indication. The indications with the highest number of reference price regulated medication packs are CARD, MUSCO, and NEURO. Highest co-payment costs—and with that highest pack prices—were found in the indications ONCIM, HAEM, and INFEC. The indications with the highest import numbers were ONCIM, HAEM, and RESP.

Research findings offer an extensive overview of the current price setting and price dynamics in Germany’s pharma market and could incentivise stakeholders to discuss current pricing policies and modify regulations and laws that are currently in force, if needed.

## Supplementary Information

**Additional file 1. **Pie chart of investigated indication areas.

**Additional file 2. **Distribution of investigated pharma companies.

**Additional file 3. **ATC indication areas with single-digit letter code and indication abbreviation.

## Data Availability

The datasets used and/or analysed during the current study are available from the corresponding author on reasonable request.
