# Supplementary figures and images for: Indication-wide drug pricing: Insights from the pharma market
Source: J Pharm Policy Pract. 2022 Aug 29;15:53. doi: 10.1186/s40545-022-00451-x (PMC9422096; doi:10.1186/s40545-022-00451-x)

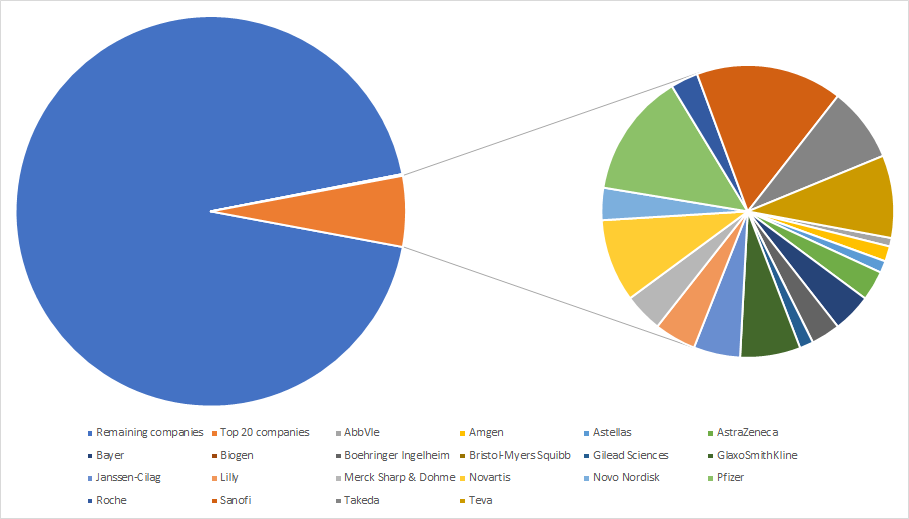

Supplement: Supplementary file 1 — Additional file 1. Pie chart of investigated indication areas. [file 40545_2022_451_MOESM1_ESM.png]

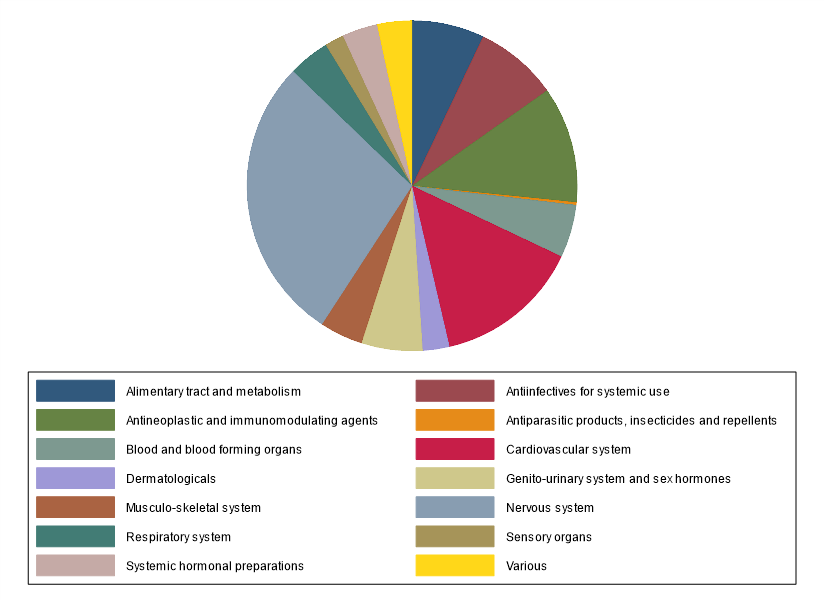

Supplement: Supplementary file 2 — Additional file 2. Distribution of investigated pharma companies. [file 40545_2022_451_MOESM2_ESM.png]

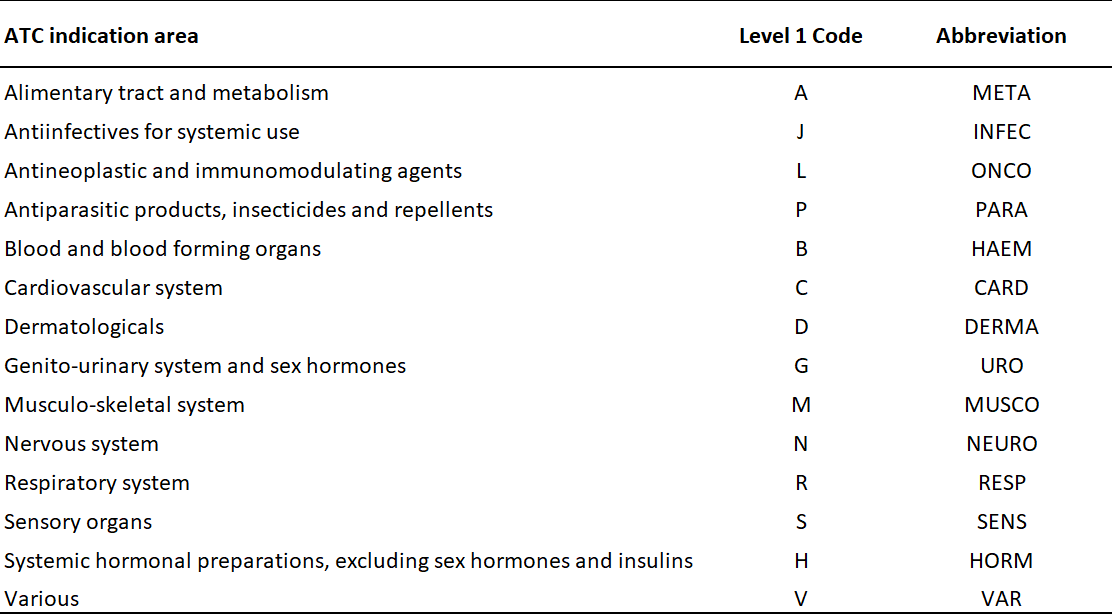

Supplement: Supplementary file 3 — Additional file 3. ATC indication areas with single-digit letter code and indication abbreviation. [file 40545_2022_451_MOESM3_ESM.png]
